# Supplementary material for: Engineered histones reshape chromatin in human cells
Source: bioRxiv. 2025 Sep 11:2025.09.10.674980. Preprint. [Version 1] doi: 10.1101/2025.09.10.674980 (PMC12439981; doi:10.1101/2025.09.10.674980)
Supplement: 1 [file NIHPP2025.09.10.674980V1-supplement-1.pdf]

bioRxiv preprint doi: https://doi.org/10.1101/2025.09.10.674980; this version posted September 11, 2025. The copyright holder for this preprint (which was not certified by peer review) is the author/funder, who has granted bioRxiv a license to display the preprint in perpetuity. It is made available under aCC-BY 4.0 International license.

Supplemental Figures

A

	H2A	H2B	H3.3	H4	total
initial UniRef search	46,150	34,392	32,876	15,569	128,987
filtered by human length	758	848	1,919	1,318	4,843
unique human-length sequences	486	550	1,218	832	3,086
mutations called	1,117	967	1,752	782	4,618

B

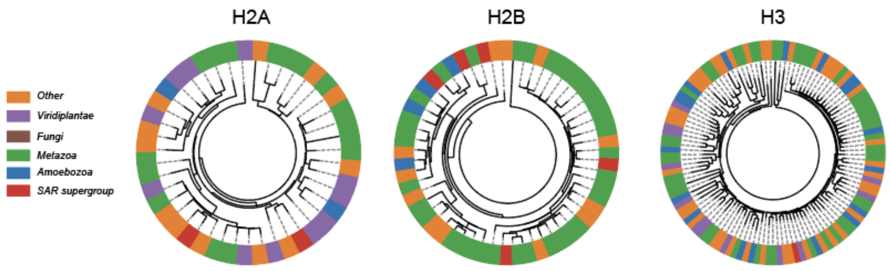

C

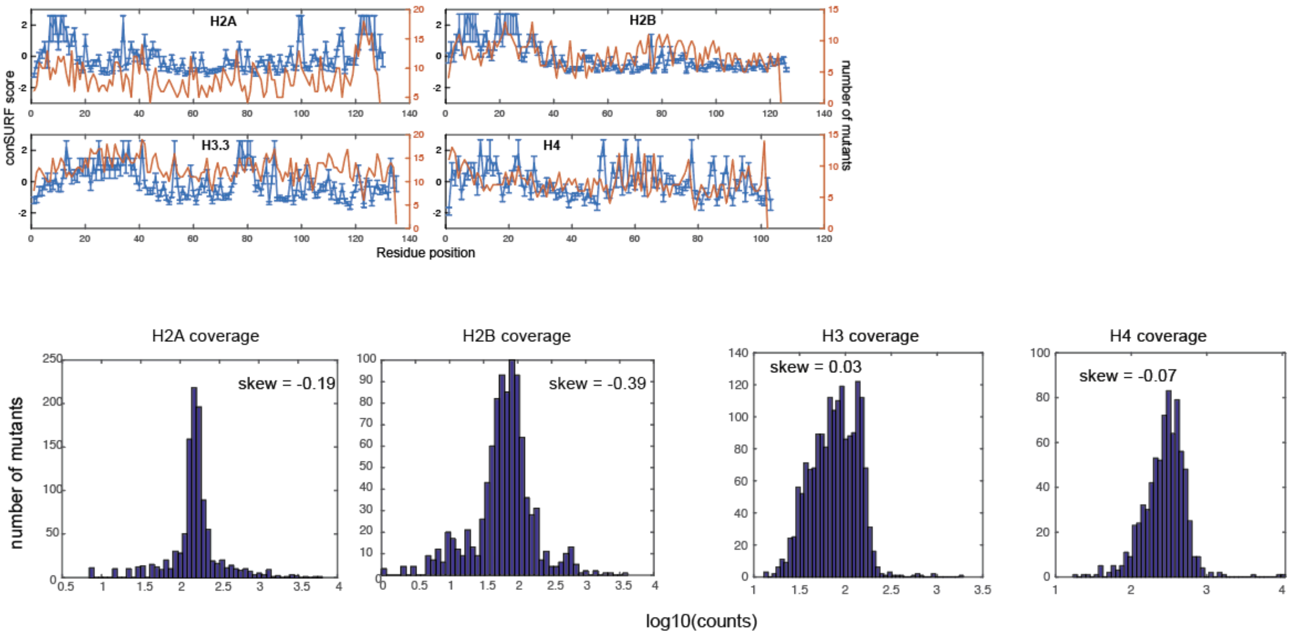

D

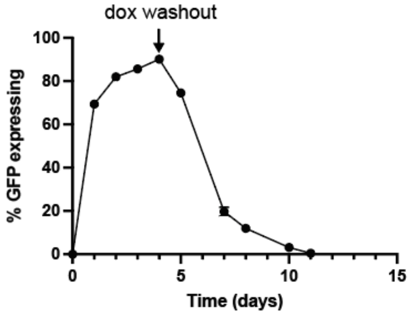

Figure S1: A. Breakdown of mutation calling steps for library creation. Histone proteins were called with UniRef, then filtered to human-length proteins, aligned, and mutations called. **B.** Phylogenetic trees for H2A, H2B, H3, constructed in the same way as H4 (Figure 1). **C. (top)** Number of mutants (orange) plotted over ConSurf conservation score (blue) **(bottom)** distribution of mutant representation in final libraries, with calculated skewness. **D.** Dynamics of induction and subsequent degradation of H4-GFP upon dox washout.

bioRxiv preprint doi: https://doi.org/10.1101/2025.09.10.674980; this version posted September 11, 2025. The copyright holder for this preprint (which was not certified by peer review) is the author/funder, who has granted bioRxiv a license to display the preprint in perpetuity. It is made available under aCC-BY 4.0 International license.

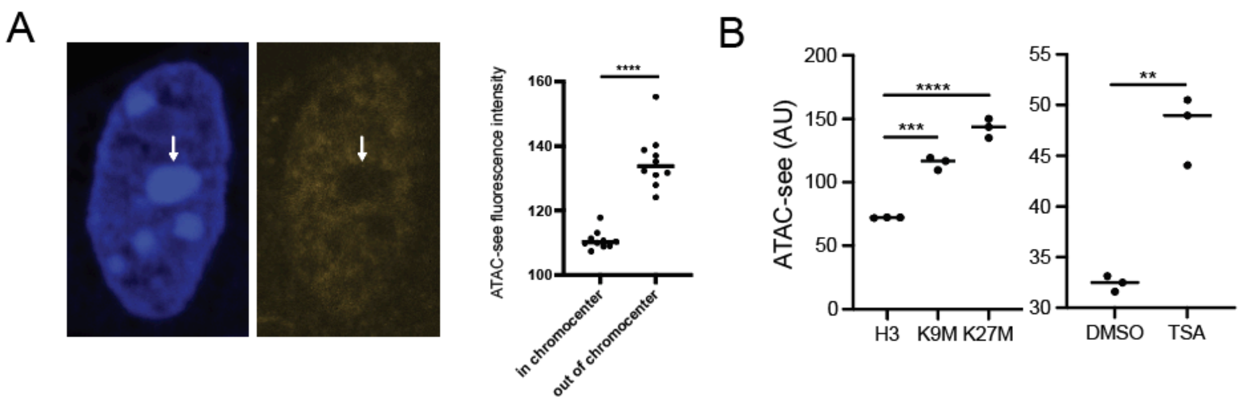

C

screen	R^2
H3 ATAC	0.89
H3 K27ac	0.96
H3 K27me3	0.91
H3 K9me3	0.87
H4 ATAC	0.88
H4 K27ac	0.91
H4 K27me3	0.92
H4 K9me3	0.93
H2A ATAC	0.88
H2B ATAC	0.70

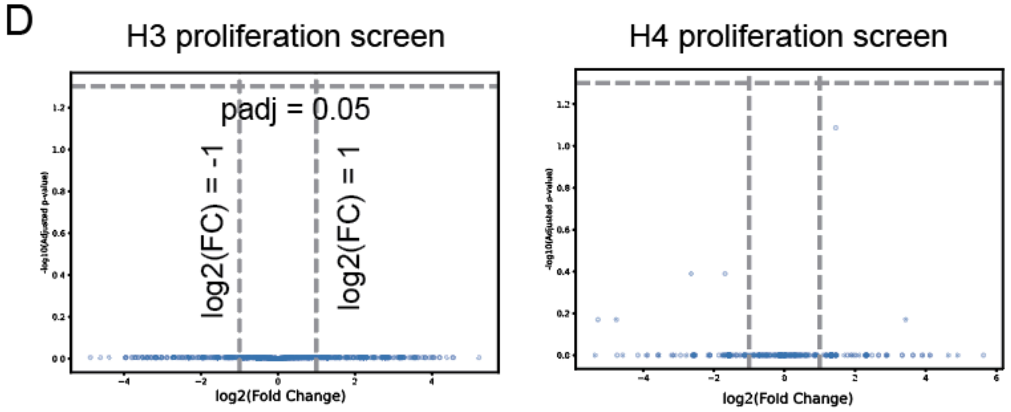

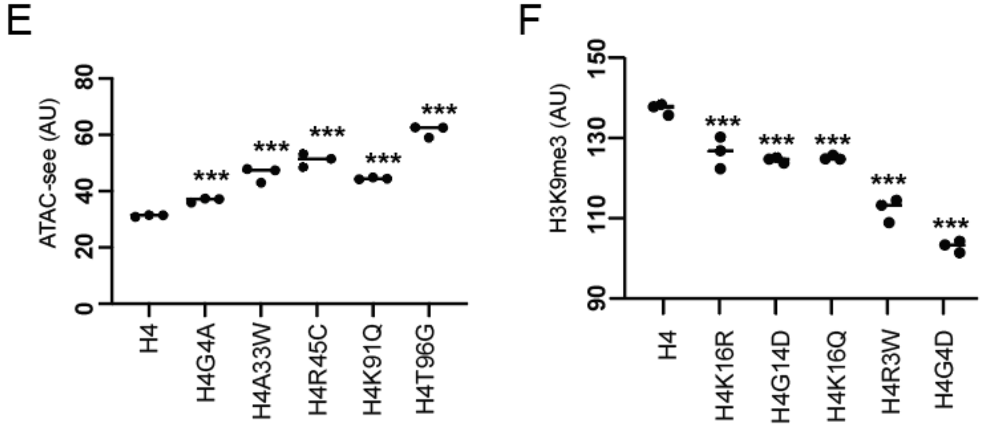

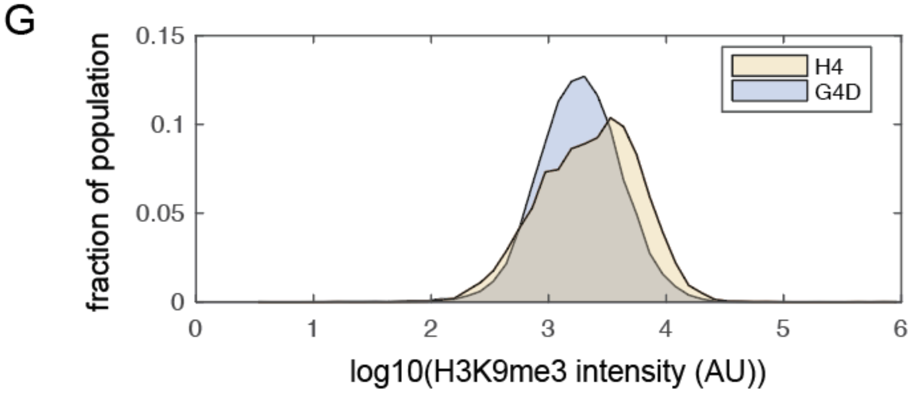

Figure S2: A. NIH-3T3 chromocenters showing high DAPI content (left) and low ATAC-see content (right), quantified on far right. **B.** H3 mutants and drugs increase accessibility of cells as measured through ATAC-see. **C.** Replicate correlation of screens in this study. **D.** H3 and H4 proliferation screens, showing no significant

bioRxiv preprint doi: https://doi.org/10.1101/2025.09.10.674980; this version posted September 11, 2025. The copyright holder for this preprint (which was not certified by peer review) is the author/funder, who has granted bioRxiv a license to display the preprint in perpetuity. It is made available under aCC-BY 4.0 International license.

effect on proliferation at the time of cell collection. **E.** Validation of selected ATAC-see hits. **F.** Validation of selected H3K9me3 hits. **G.** Example of flow data comparing G4D to H4, showing decrease (~30%) in mean H3K9me3 levels.

bioRxiv preprint doi: https://doi.org/10.1101/2025.09.10.674980; this version posted September 11, 2025. The copyright holder for this preprint (which was not certified by peer review) is the author/funder, who has granted bioRxiv a license to display the preprint in perpetuity. It is made available under aCC-BY 4.0 International license.

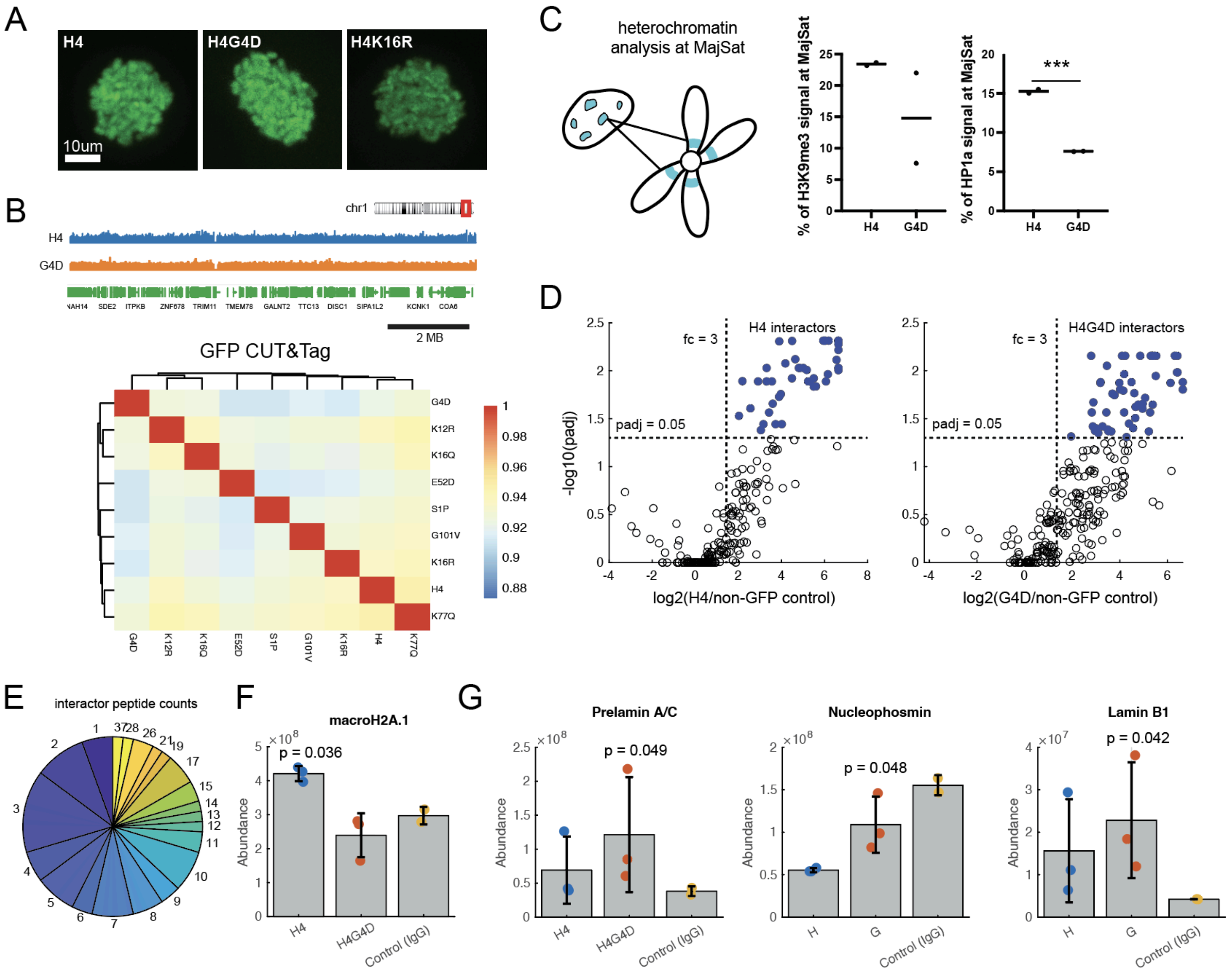

Figure S3: A. Mitotic chromatin demonstrates proper incorporation of mutant H4s. **B.** CUT&Tag of GFP-tagged histones with a range of mutations demonstrate high correlation (>0.9) between genomic enrichment levels across 50-kb bins. Sample tracks for H4 and G4D shown for a randomly selected region of chr1. **C.** CUT&Tag of H3K9me3 and HP1a in Major Satellite repeats in NIH-3T3s demonstrates a possible but not significant decrease in H3K9me3 (left) and a significant decrease of HP1a at MajSat sites (right). *** denotes $p < 0.0005$. **D.** Defining interactors of H4-GFP and H4G4D-GFP using RIME, by adjusted p-value cutoff and foldchange. **E.** Representative peptides per interactor, with most interactors represented by > 2 unique peptides. **F-G** Protein abundance for macroH2A.1, Prelamin A/C, Nucleophosmin, and Lamin B1, measured using RIME.

bioRxiv preprint doi: https://doi.org/10.1101/2025.09.10.674980; this version posted September 11, 2025. The copyright holder for this preprint (which was not certified by peer review) is the author/funder, who has granted bioRxiv a license to display the preprint in perpetuity. It is made available under aCC-BY 4.0 International license.

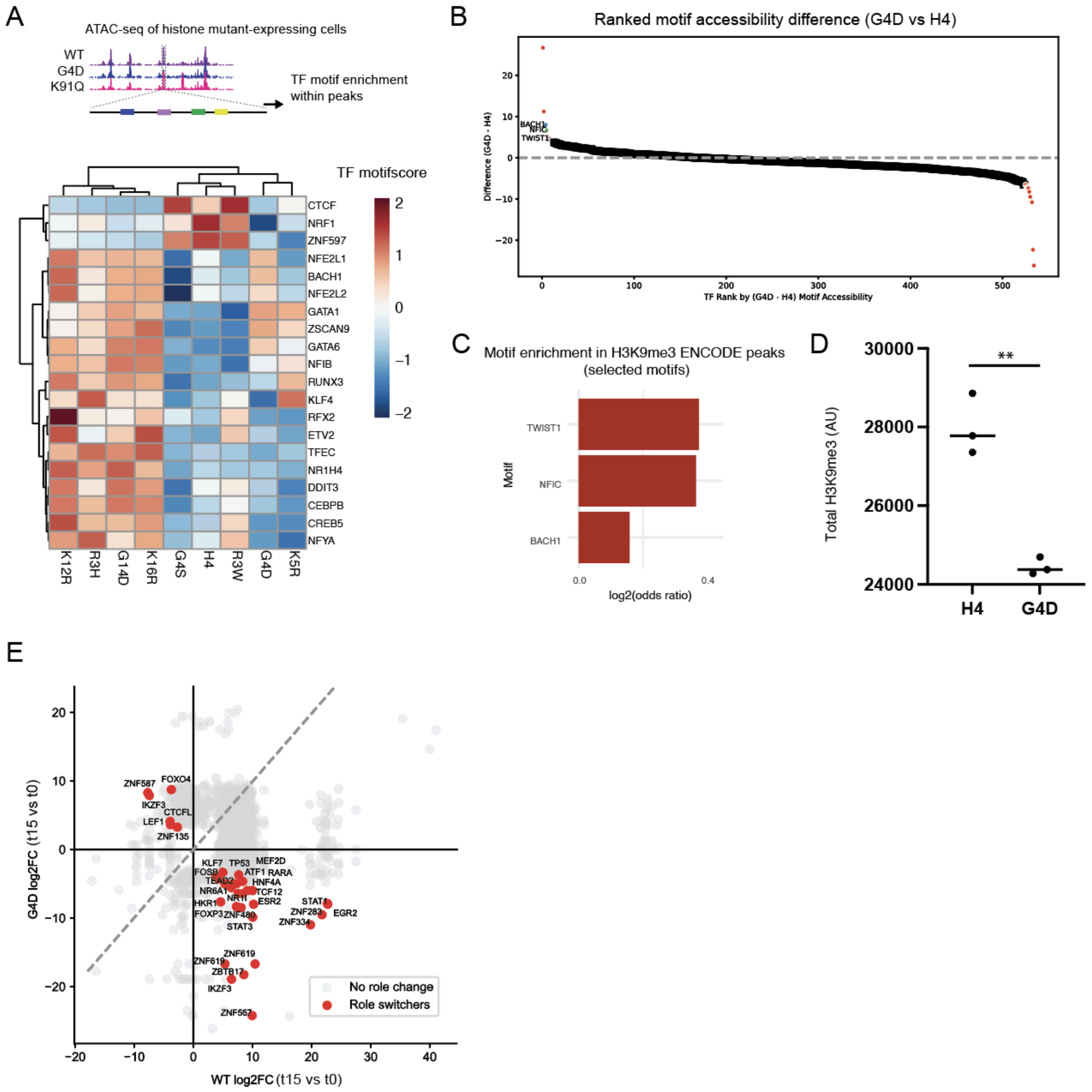

Figure S4: A. Schematic showing ATAC-seq workflow and enrichment (TF motifscore) of the most variable TF motifs in K562s. **B.** Ranked plot of motif accessibility difference in G4D vs H4 K562s. **C.** Enrichment of motifs demonstrating high accessibility increase in H3K9me3 peaks. **D.** Decrease in H3K9me3 in Jurkat T cells expressing G4D. **E.** Scatter plot of transcription factor effects on G4D vs H4 K562s, with role-switchers (significant (padj < 0.05) hits off of the diagonal) highlighted in red.

bioRxiv preprint doi: https://doi.org/10.1101/2025.09.10.674980; this version posted September 11, 2025. The copyright holder for this preprint (which was not certified by peer review) is the author/funder, who has granted bioRxiv a license to display the preprint in perpetuity. It is made available under aCC-BY 4.0 International license.

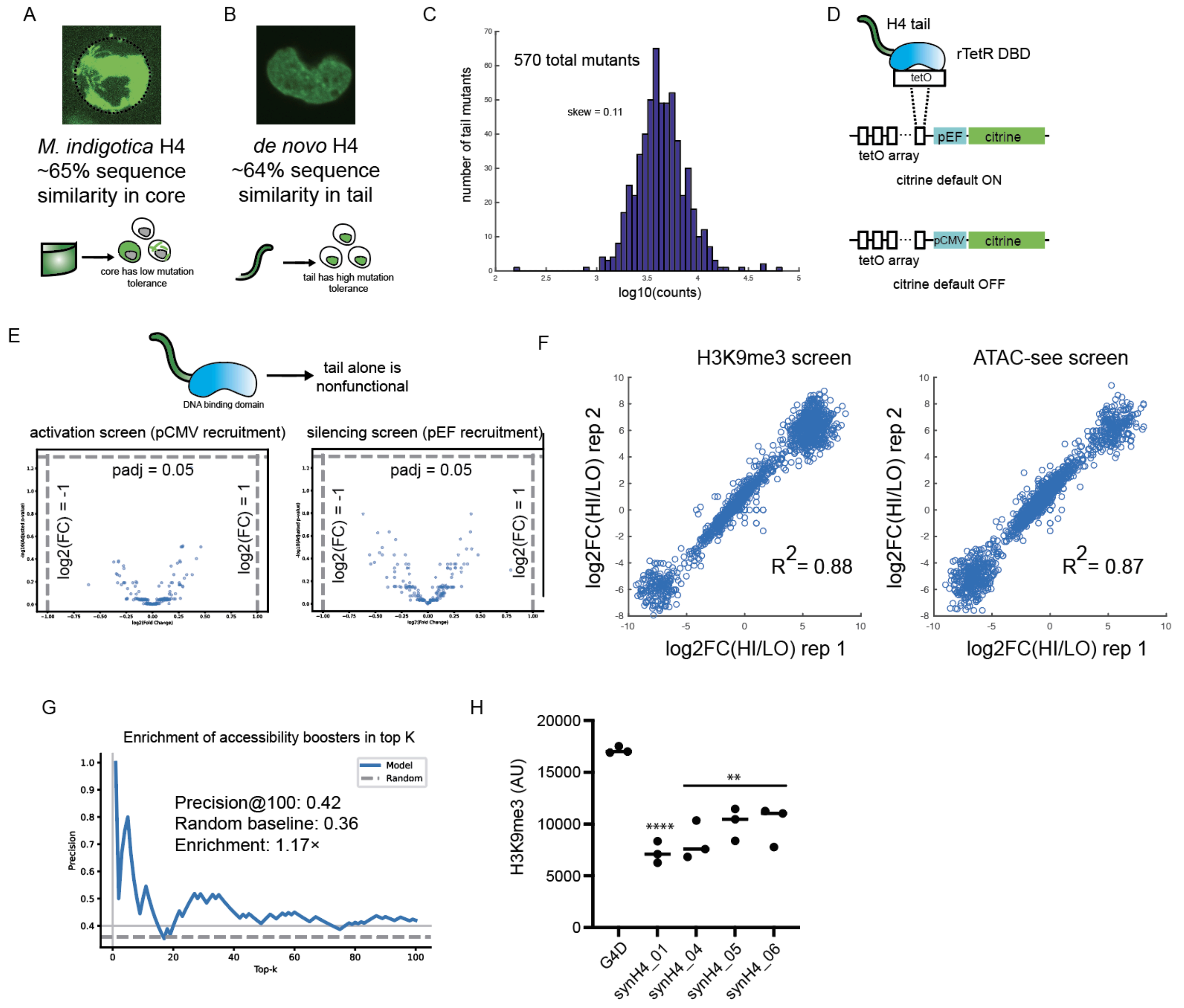

Figure S5: Mutations of the core (**A**) but not the tail (**B**) of H4 cause mislocalization and loss of function of the protein. **C.** Distribution of tail mutant representation over 570 total mutants, with calculated skewness, in HT-RECRUIT screens. **D.** activation and silencing HT-RECRUIT screen overview. **E.** HT-recruit screens showing lack of activity of tails alone. **F.** Replicate correlation of double-mutant H3K9me3 (left) and ATAC-see (right) screens. **G.** Precision@k plot for H4 tails that increase ATAC-see score, using the histForge model. **H.** Decrease in H3K9me3 levels in Jurkat cells expressing synH4 constructs.
